# Effects of N-Acetylcysteine combined with Ambroxol Hydrochloride on clinical symptoms, CRP, and PCT in children with pneumonia

**DOI:** 10.1016/j.clinsp.2024.100476

**Published:** 2024-08-28

**Authors:** AiLi Xue, Hua Zhang, ShanShan Song, Xia Yu

**Affiliations:** a Department of Infants, Women and Children's Hospital, Qingdao University, Qingdao City, Shandong Province, China; b Department of Operating Theatre, Qingdao Central Hospital, Qingdao City, Shandong Province, China; c Department of Paediatrics, Binzhou Maternal and Child Health Hospital, Binzhou City, Shandong Province, China

**Keywords:** Pediatric pneumonia, N-acetylcysteine, Ambroxol hydrochloride, Combination therapy, Clinical symptoms, C-reactive protein, Procalcitonin

## Abstract

**Objective:**

This study investigated the effects of N-Acetylcysteine (NAC) combined with Ambroxol Hydrochloride (AH) on clinical symptoms, C-Reactive Protein (CRP), and Procalcitonin (PCT) levels in children with pneumonia.

**Methods:**

A total of 98 children with pneumonia were assigned to the control group and observation group by random number table method. NAC was administered to the observation group and AH was given to the control group. The therapeutic effect was observed, the disappearance time of clinical symptoms and levels of inflammatory factors, lung function parameters, blood gas analysis parameters, and immunoglobulin were measured. The incidence of adverse reactions was statistically analyzed.

**Results:**

A higher effective rate was observed in the observation group than in the control group (*p* < 0.05). Antipyretic time, cough disappearance time, and lung rale disappearance time in the observation group were shorter than those in the control group (*p* < 0.05). After treatment, CRP and PCT were lower (*p* < 0.05), FVC, FEV1, and FEV1/FVC were higher, PaCO_2_ was lower, PaO_2_ and SaO_2_ were higher, and IgA, IgG, IgM, and C3 were higher in the observation group than those in the control group (*p* < 0.05). The incidence of adverse reactions between the two groups was not significantly different (*p* > 0.05).

**Conclusion:**

NAC combined with AH is effective in the treatment of pediatric pneumonia by effectively alleviating clinical symptoms, reducing inflammatory factors, and improving lung function and immune function.

## Highlights

• NAC combined with AH is effective in treating pneumonia.
• NAC combined with AH alleviates clinical symptoms of pneumonia.
• NAC combined with AH reduces inflammatory response in pneumonia.
• NAC combined with AH improves blood gas and immune function in pneumonia.
• NAC combined with AH improves lung function in pneumonia.

## Introduction

Pneumonia is a common pediatric disease clinically manifested as fever, cough, shortness of breath, and medium-fine moist lung rale. It is often caused by bacterial infection and is mainly treated by antibiotics. However, antibiotics have toxic effects on the nervous system, blood system, liver, and kidney organs, and long-term use will lead to liver and kidney function impairment. Symptomatic treatments for pediatric pneumonia currently include Ambroxol Hydrochloride (AH) and N-Acetylcysteine (NAC) atomized liquid.^1^ Data have demonstrated that both AH and NAC atomized liquid can promote mucus elimination, and NAC can also act as an oxidizing agent to interfere with the generation of free radicals, and the combination of the two can achieve the expected therapeutic effect on bronchopneumonia in children.^2^ In the treatment of pediatric pneumonia, NAC atomized solution can be used in combination with AH to resolve phlegm and improve lung symptoms.^3^ Based on this, this study aims to explore the effects of NAC combined with AH in the treatment of pediatric pneumonia regarding the regulation of clinical symptoms, C-Reactive Protein (CRP), and Procalcitonin (PCT) levels.

## Data and methods

### *Clinical data*

A total of 98 children with pneumonia from January 2021 to January 2023 were assigned to the control group and observation group by random number table method. Inclusion criteria: 1) Patients meeting complied with the diagnostic criteria for pneumonia;^4^ 2) Patients between the ages of 2 and 6 years, Asian; 3) Under the approval of the Ethics Committee of Women and Children's Hospital, Qingdao University (QFELL-YJ-2024-70), patients gave informed consent; 4) Patients with fever, cough, and wheezing; 5) Patients with well drug tolerance. Exclusion criteria: 1) Patients combined with pleural effusion or empyema; 2) Patients with pulmonary tuberculosis and other respiratory diseases (Combined bronchial asthma, Congenital lung cysts, Pulmonary hypoplasia, etc.); 3) Patients with abnormal growth or severe malnutrition; 4) Patients combined with severe liver and kidney disease, systemic infection, immune deficiency, and congenital heart disease; 5) Patients with drug allergy or drug incompatibility; 6) Patients combined with viral encephalopathy; 7) Patients combined with hemolytic uremic syndrome. This clinical observational study follows the STROBE statement.

## Methods

Fluid therapy and symptomatic treatment were given to the children in both groups to alleviate coughing, dissipate phlegm, and subside fevers. The control group was treated with AH (Shandong Luoxin Pharmaceutical Group STOCK Co., Ltd., H20051402, specifications: 300 mg/tablet). One tablet of AH was dissolved in 3 mL 0.9 % sodium chloride solution, added to the mask atomizer, and connected to the oxygen device with an adjusted oxygen flow rate of 6‒8 L/min. Atomized inhalation was performed twice a day for 1 week.

The observation group was given combined treatment with NAC (ZAMBONS.R.L, H20110405, specification: 3 mL:300 mg). NAC (3 mL) was dissolved in 3 mL 0.9 % sodium chloride solution, and other procedures in atomized inhalation of NAC were performed as same as AH inhalation.

### Observation indicators

(1) Clinical effect:^5^ 1) Cure: clinical symptoms such as fever, cough, shortness of breath, and lung rales completely disappeared, and laboratory indicators such as lung X-Ray film and blood routine returned to normal; 2) Effective: clinical symptoms disappeared, and laboratory indicators did not return to normal; 3) Ineffective: no improvement in clinical symptoms after 3 days of medication. Total effective rate = cure rate + effective rate.
(2) Disappearance time of clinical symptoms: antipyretic time, cough disappearance time, and lung rales disappearance time were compared.
(3) Inflammatory factors: CRP and PCT were determined by ELISA.
(4) Lung function indicators: Force Vital Capacity (FVC) and Force Expiratory Volume one Second (FEV1) were monitored by the Jaeger MasterScreen Pneumo.
(5) Blood gas analysis: Elbow arterial blood samples (6 mL) was collected from each patient in both groups after morning fasting and placed in heparin anticoagulation tubes, and arterial Partial Pressure of Oxygen (PaO_2_), Arterial Carbon Dioxide Partial Pressure (PaCO_2_), and blood Oxygen Saturation (SaO_2_) were detected by Siemens Rapidpoint348 blood gas analyzer. Analysis was performed by the same physician.
(6) Immunoglobulin level: Peripheral venous blood (20 mL) was extracted from each patient using a fully automatic biochemical analyzer of BoKO BKG1200, and IgM, IgA, IgG, and C3 levels were detected by immunoturbidimetry using a fully automatic biochemical analyzer (BKG1200, BIOBASE, Shandong, China).

### *Statistical analysis*

SPSS 22.0 software was utilized to process the data, the enumeration data were expressed as percentages and evaluated by χ^2^ test, and the measurement data were expressed as (x ± s) after the normality test and compared by *t*-test. The difference was statistically significant at *p* < 0.05.

## Results

### *Clinical data*

Clinical data between the two groups showed no statistical significance (*p* > 0.05, Table 1).

**Table 1:** Comparison of clinical data between the two groups.

**Parameters**	**Observation group**	**Control group**	***χ*^2^/*t***	**p**
	**(*n*=49)**	**(*n*=49)**		
Gender			0.169	0.681
Male	28	30		
Female	21	19		
Age (years)	5.28 ± 1.03	5.34 ± 1.12	0.276	0.783
Course pf disease (days)	5.16 ± 0.57	5.29 ± 0.64	1.062	0.291
Body weight (kg)	8.95 ± 1.78	9.03 ± 1.29	0.255	0.799
WBC (*10^9^/L)	15.01 ± 2.01	15.21 ± 2.16	0.474	0.636

### *Clinical effect*

The observation group had a higher total effective rate than the control group (*p* < 0.05, Table 2).

**Table 2:** Comparison of therapeutic effect between two groups (case, %).

**Groups**	**n**	**Cure**	**Effective**	**Ineffective**	**Total effective rate**
Observation group	49	19	28	2	95.92
Control group	49	15	26	8	83.67
*χ*^2^					4.009
P					0.045

### *Disappearance time of clinical symptoms*

**Fig. 1:**
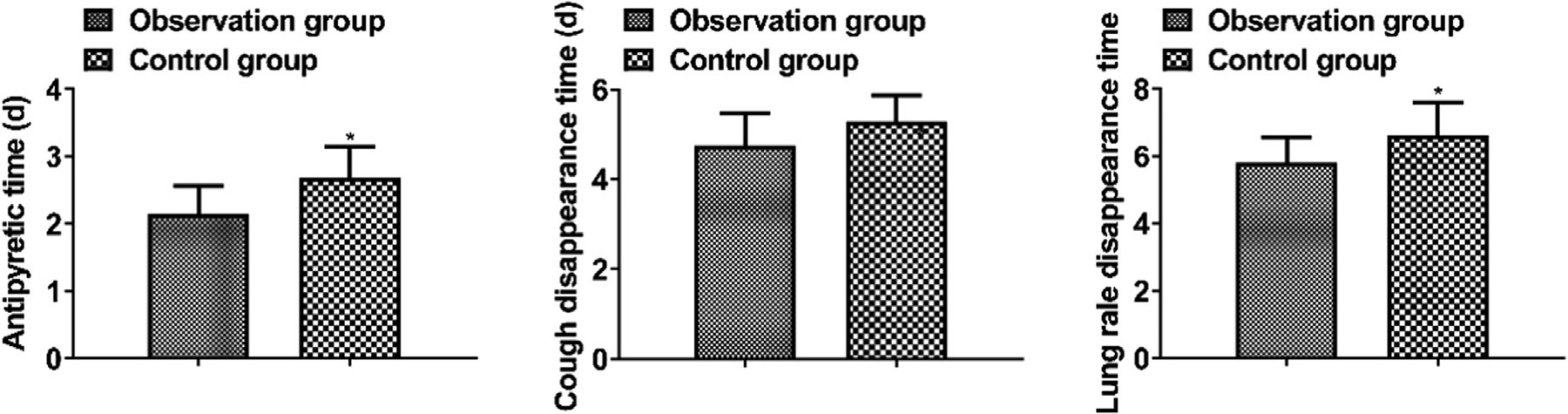
Disappearance time of clinical symptoms.

The antipyretic time, cough disappearance time, and lung rale disappearance time in the observation group were shorter than those in the control group (*p* < 0.05, Fig. 1).

### *Inflammatory factors*

**Fig. 2:**
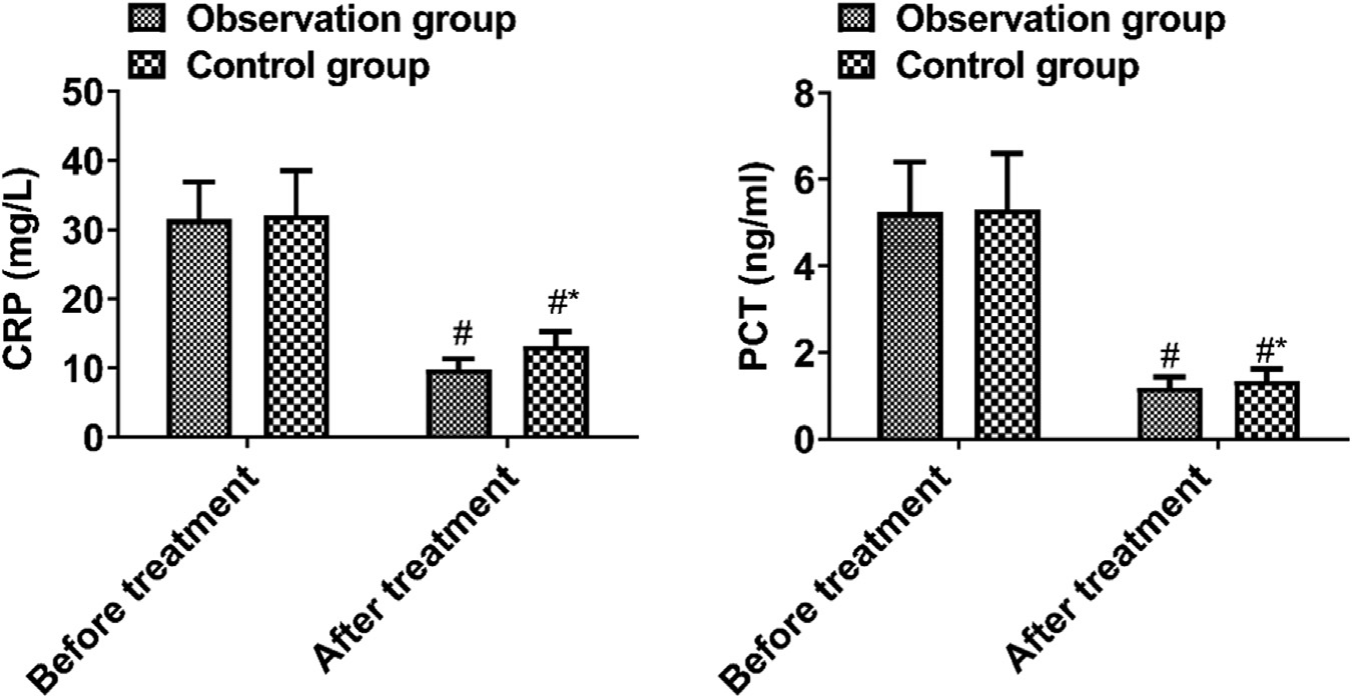
Inflammatory factors.

Pre-treatment inflammatory factors were not significantly different between the two groups (*p* > 0.05). As compared with the control group, the observation group had lower CRP and PCT levels after treatment (*p* < 0.05, Fig. 2).

### *Lung function*

**Fig. 3:**
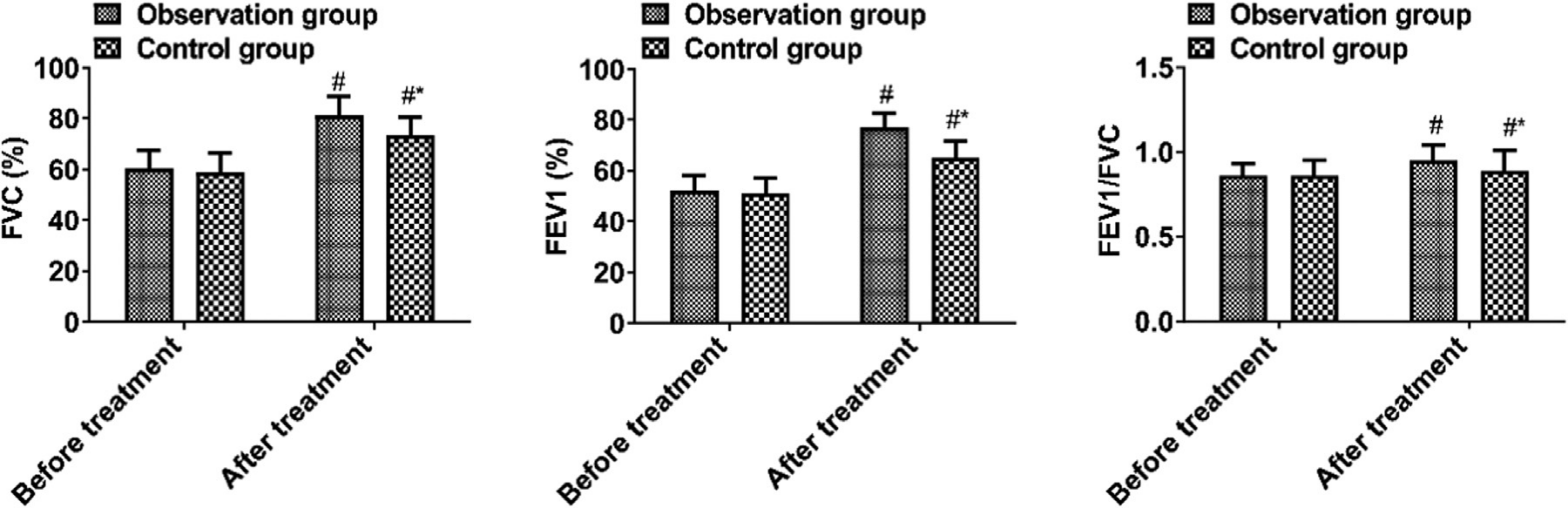
Lung function parameters.

Before treatment, there was no significant difference in pulmonary function parameters between the two groups (*p* > 0.05). There were higher values of FVC, FEV1, and FEV1/FVC in the observation group than in the control group after treatment (*p* < 0.05, Fig. 3).

### *Blood gas analysis*

**Fig. 4:**
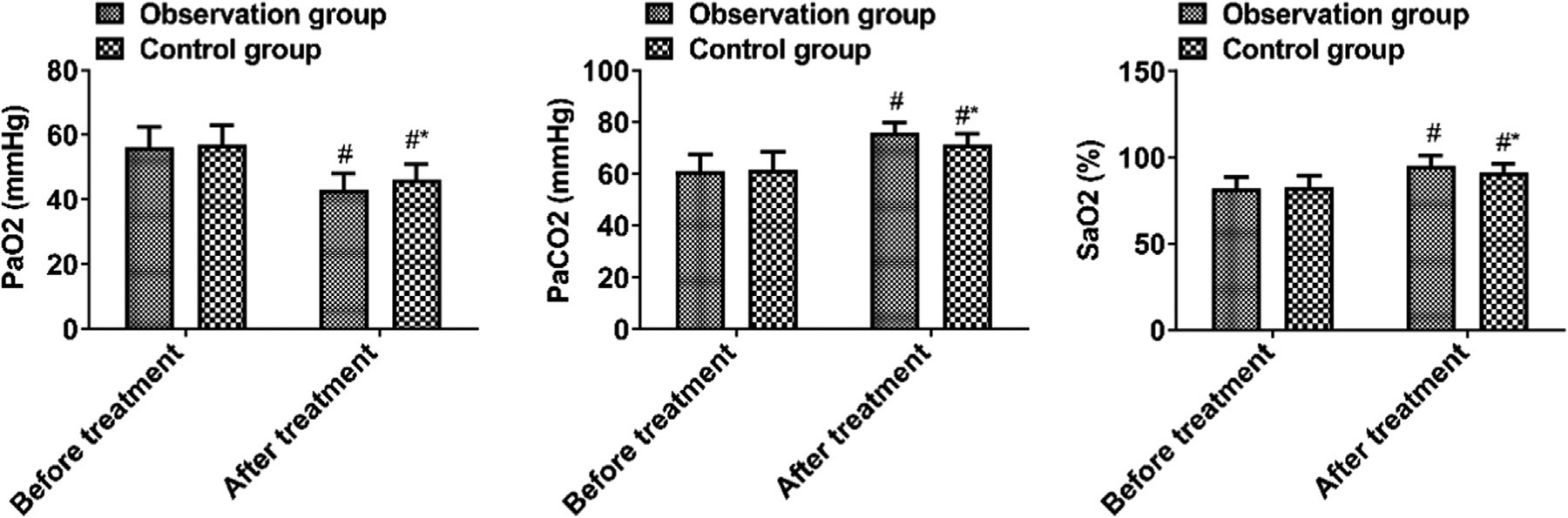
Blood gas analysis parameters.

Before treatment, there were no significant differences between the two groups in blood gas analysis parameters (*p* > 0.05). After treatment, PaCO_2_ was lower, while PaO_2_ and SaO_2_ were higher in the observation group than in the control group (*p* < 0.05, Fig. 4).

### *Immunoglobulin levels*

**Fig. 5:**
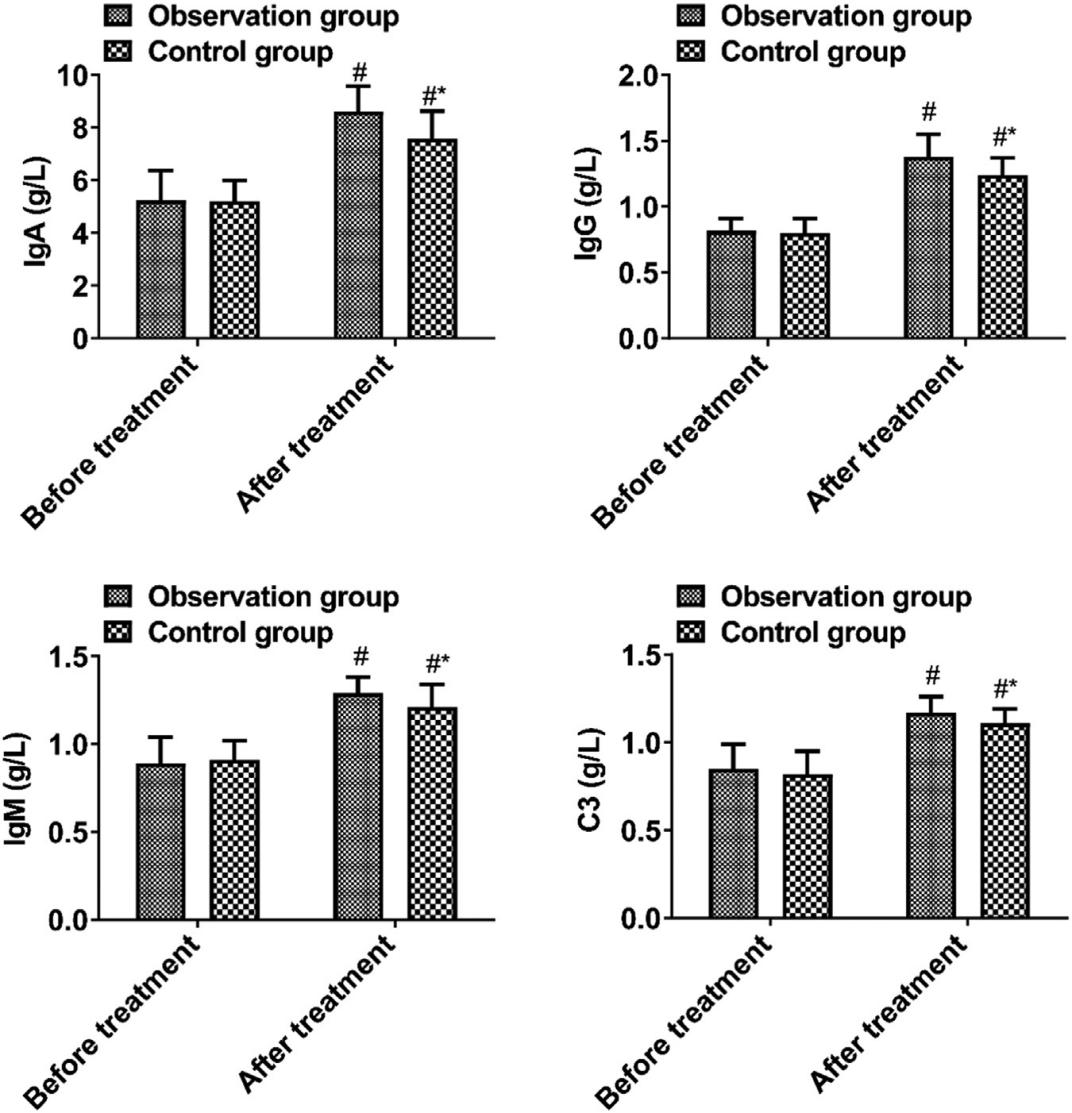
Immunoglobulin level. Note: Compared with the group before treatment, # *p* < 0.05; Compared with observation group after treatment, * *p* < 0.05.

Before treatment, no significant difference was observed in immunoglobulin levels between the two groups (*p* > 0.05). The observation group had higher levels of IgA, IgG, IgM, and C3 after treatment than the control group (*p* < 0.05, Fig. 5).

### *Adverse reactions*

Adverse reactions were not significantly different between the two groups (*p* > 0.05, Table 3).

**Table 3:** Comparison of adverse reactions between the two groups (cases, %).

**Groups**	**n**	**Nausea and vomiting**	**Pruritus**	**Celialgia**	**Total incidence**
Observation group	49	1	0	2	6.12
Control group	49	1	1	0	4.08
*χ*^2^					0.211
P					0.646

## Discussion

Children are susceptible to infection and pneumonia because their lung tissue and immune function are not fully mature and their ability to resist pathogens is poor. The severity of pneumonia in children varies greatly, and the progression of the disease involves multiple organs or systems, induces hypercapnia and hypoxemia, etc., which seriously affect the growth and development of children and even endanger their life safety.^6^ Therefore, it is particularly important to actively explore the treatment methods to alleviate the disease quickly. AH can alleviate the clinical symptoms of pediatric pneumonia. However, according to relevant reports, the clinical efficacy of single-drug therapy is poor, so multi-drug combination therapy is needed.^7^^,^^8^ NAC, as a new mucous solvent, can gradually reduce the adhesion ability of phlegm, improve sputum discharge, and reduce the inflammatory stimulation of the respiratory tract. Moreover, NAC has a certain inhibitory effect on polyglycoprotein and effectively reduces the damage effect of bacteria on cell membranes based on the inhibition of bacterial adhesion ability.^9^^,^^10^ In this study, the treatment effective rate was higher, the disappearance time of clinical symptoms was shorter, FVC, FEV1, FEV1/FVC, PaO_2,_ and SaO_2_ were higher, and PaCO_2_ was lower in the observation group than those of the control group. It shows that NAC combined with AH is effective in treating pneumonia in children, which can effectively reduce clinical symptoms and improve lung function. This is mainly because NAC atomized solution is a powerful phlegm-dissolving agent, which can not only dissolve mucus sputum but also treat porous sputum caused by bacterial infection. A combination of NAC atomized solution and AH can effectively alleviate bronchopneumonia symptoms in children by dissolving phlegm and improving lung function.^11^

Clinical data show that NAC also regulates the balance of the immune system, promotes the synthesis and secretion of immune proteins and complement, inhibits the production of inflammatory factors, greatly alleviates inflammatory response, and thus improves clinical symptoms and shortens disease course.^12^^,^^13^ In this study, combined treatment reduced the inflammatory response of the body by suppressing CRP and PCT. The reason is that NAC is a precursor of intracellular GSH, which can effectively reduce the active substance in the bronchial alveolar cavity and plasma of patients.^14^ According to relevant reports, NAC is also an antioxidant, which/effectively removes reactive oxygen species. By inhibiting toxins in the body, it can effectively block the NB-kB pathway, reduce IL-8 and IL-6 expression, and achieve the purpose of inhibiting inflammation.^15^^,^^16^

Mucus from infections often blocks the trachea in children due to tracheal stenosis, poor lung elastic tissue development, and a small number of alveoli. At the same time, the immune system of children has not been fully established and improved, and cannot effectively resist the invasion of pathogenic bacteria, so children are prone to pneumonia.^17^ However, pneumonia pathogens not only cause local inflammatory damage to respiratory mucosa but also activate the immune response of the body and induce immune damage. It has been proposed that cellular and humoral immunity disorders still exist in most children after treatment with AH.^18^ In this study, NAC combined therapy improved the immune function of pediatric pneumonia patients by elevating IgA, IgG, IgM, and C3. The reason is that the effective components of NAC interfere with the regulation of T lymphocyte subgroup function by inflammatory cytokines and improve immune function indicators.^19^^,^^20^ Moreover, NAC with AH would not increase adverse reactions, that is, the combination treatment was relatively safe.

In summary, NAC combined with AH is highly effective in the treatment of pediatric pneumonia, which can effectively reduce clinical symptoms, reduce the level of inflammatory factors in the body, and improve the lung function and immune function of patients.

## Ethics approval and consent to participate

This study was reviewed and approved by the Medical Ethics Committee of Women and Children's Hospital, Qingdao University (QFELL-YJ-2024-70), and all subjects gave informed consent.

## Authors’ contributions

Conceptualization, AiLi Xue; Methodology, AiLi Xue and Hua Zhang; Formal analysis, Hua Zhang and ShanShan Song; Investigation, Hua Zhang and ShanShan Song; Data curation, Xia Yu; Writing-original draft preparation, AiLi Xue; Writing-review and editing, Xia Yu; Project administration, Xia Yu. All authors have read and agreed to the published version of the manuscript.

## Funding

Not applicable.

## Declaration of competing interest

The authors declare no conflicts of interest.
